# CrossNorm: a novel normalization strategy for microarray data in cancers

**DOI:** 10.1038/srep18898

**Published:** 2016-01-06

**Authors:** Lixin Cheng, Leung-Yau Lo, Nelson L. S. Tang, Dong Wang, Kwong-Sak Leung

**Affiliations:** 1 Department of Computer Science and Engineering, The Chinese University of Hong Kong, Shatin, New Territories, Hong Kong; 2 Department of Chemical Pathology, The Chinese University of Hong Kong, Shatin, New Territories, Hong Kong; 3 College of Bioinformatics Science and Technology, Harbin Medical University, Harbin, China

## Abstract

Normalization is essential to get rid of biases in microarray data for their accurate analysis. Existing normalization methods for microarray gene expression data commonly assume a similar global expression pattern among samples being studied. However, scenarios of global shifts in gene expressions are dominant in cancers, making the assumption invalid. To alleviate the problem, here we propose and develop a novel normalization strategy, Cross Normalization (CrossNorm), for microarray data with unbalanced transcript levels among samples. Conventional procedures, such as RMA and LOESS, arbitrarily flatten the difference between case and control groups leading to biased gene expression estimates. Noticeably, applying these methods under the strategy of CrossNorm, which makes use of the overall statistics of the original signals, the results showed significantly improved robustness and accuracy in estimating transcript level dynamics for a series of publicly available datasets, including titration experiment, simulated data, spike-in data and several real-life microarray datasets across various types of cancers. The results have important implications for the past and the future cancer studies based on microarray samples with non-negligible difference. Moreover, the strategy can also be applied to other sorts of high-throughput data as long as the experiments have global expression variations between conditions.

Gene microarrays have been commonly used for global expression analysis of biological systems^1^,^2^,^3^. Moreover, normalization is widely regarded as an essential step before the microarray data analysis, in order to remove systematic experimental bias and technical variation while maintaining biological signals of interest^4^. The choice of normalization method has a profound impact on gene expression estimates^5^. Essentially, the results obtained by methodologies based on distinct assumptions could lead to entirely different biological interpretations, which call for development of more robust and effective normalization methods^6^. Currently, most normalization methods make two basic assumptions about the data, which are 1) only a few genes are over-expressed or under-expressed in one array relative to the others, and 2) the number of genes over-expressed in a condition is similar to the number of genes under-expressed^4^,^6^,^7^,^8^. Both of the two assumptions should agree with the experimental context when applying the corresponding methodologies. If the expression levels of all genes are globally equivalent or similar over the arrays, then normalized expression data should produce an accurate representation of the relative levels of each gene product. Otherwise, methodologies on the basis of the two basic assumptions may fail to produce biologically meaningful interpretation. Previous studies have found that genes are widely up-regulated and tend to have variable expression in a variety of cancers microarray datasets^6^,^7^. Furthermore, Lin, C.Y. *et al.* recently found transcriptional amplification in tumor cells with elevated c-Myc level. Cells with high levels of c-Myc can amplify their gene expression programs, producing two to three times more total RNA than their low-Myc counterparts^9^,^10^. In these scenarios, the differential expression of genes is predominately in one direction and hence a great number of genes are differentially expressed between cancer and normal states. All of these discoveries have led us to challenge loads of previous works that assume genes express evenly among arrays without allowing for transcriptional amplification or repression. Simply put, it is unreasonable to expect all genes to have similar distributions with respect to the expression levels of samples in different biological groups (e.g., normal and cancer states).

However, virtually all well-accepted conventional normalization methods, such as Quantile, Baseline and LOESS normalization^11^,^12^,^13^, rely on the strong assumptions and perform poorly when the processing data are far from the assumptions, e.g., comparison of genes from cancer and normal states. More specifically, it is usual to normalize microarray data by forcing all of the arrays to have the same/similar distributions of probe intensity to remove technical variations in the data, such as the leading stochastic-model-based procedure, Quantile normalization, which even assigns an identical expression distribution for all arrays based on the rank of the measured intensity relative to all other probes on the array. Misinterpretation of microarray expression data is quite prevalent due to misunderstanding or abuse of the common assumptions, or just automatically using these methods without any pre-analysis. In particular, Quantile normalized microarray datasets were usually applied in cancer studies and were provided for other researchers, such as GSE15471 and GSE16515 for pancreatic cancer^14^,^15^ as well as GSE20347 and GSE23400 for esophageal squamous cell carcinoma^16^,^17^. Because lack of robust normalization methods for expression data, practically all the experiments available in Gene Expression Omnibus (GEO)^18^ just simply employ the conventional normalization algorithms according to the basic assumptions.

Recently, the realization that current methods may lead to erroneous biological interpretation of transcriptome experiments have boosted the proposal of several methods, e.g. LVS and NVSA^19^,^20^. These tools are rarely used, however, partially due to their limitations. For instance, the LVS algorithm^19^ requires pre-selection of a proportion (40-60%) of genes as a reference set. But it engenders some problems of its own: we cannot conceive the exact number of genes changing in one state for a real-life experiment. It has a high risk of over fitting the data if the ideal reference gene set is arbitrarily chosen. Another algorithm, NVSA, may mistreat the variant genes as invariants when the percentage of variant genes is greater than 50%^20^. The choice of the bin width defined as fixed-width intervals of expression intensity is also a potential factor affecting the performance of NVSA, as it is too arbitrary and sensitive for data with dissimilar properties. Accumulated evidence suggests that the biological variation might be greater than the system variation introduced by technical noise in the microarray datasets^6^,^7^,^21^,^22^, which leads us to explore the information from the raw data with proper methods instead of destroying it. Hence, we have developed a novel normalization strategy, Cross Normalization (CrossNorm), for microarray datasets with global shift and unbalanced variation. It makes use of the overall statistics of the original signals for all samples and the results show significantly improved robustness and accuracy in estimating transcript level dynamics for a series of publicly available datasets, involving titration experiment, simulated data, spike-in data and several real-life microarray datasets across various types of cancers.

In the following sections, firstly, we show how the algorithm-driven artifact is generated in the step of normalization, confirming and extending the finding that conventional normalization consistently overestimates sample similarity. After that, we show that CrossNorm is more robust in producing accurate assessments of transcript changes between samples in simulated data, spike-in data, titration experiment and several real sample-paired cancer datasets, respectively. We then demonstrate that CrossNorm outperforms the conventional methods from the point of expression direction that we consistently stressed. Finally, we show that the two versions of CrossNorm (Pairwise and General) perform comparably to each other and thereby the method is also applicable to more general non-paired experiments.

## Results

### Global shift exists in cancer expression data

Previous results^6^,^7^ illustrate that genes tend to be extensively up-regulated in cancers in comparison with matched normal tissue in most of cancer datasets. Specifically, the raw signal intensities in cancer samples tend to be significantly or marginally significantly higher among more than half of the cancer datasets. The percentages even increased further to 80% when focused on five larger datasets with statistically sufficient sample size (≥70). Hence, it demonstrates that the distribution of probe intensity is dissimilar for different biological condition and accordingly the common assumptions for normalization are not suitable for these scenarios anymore.

**Figure 1:**
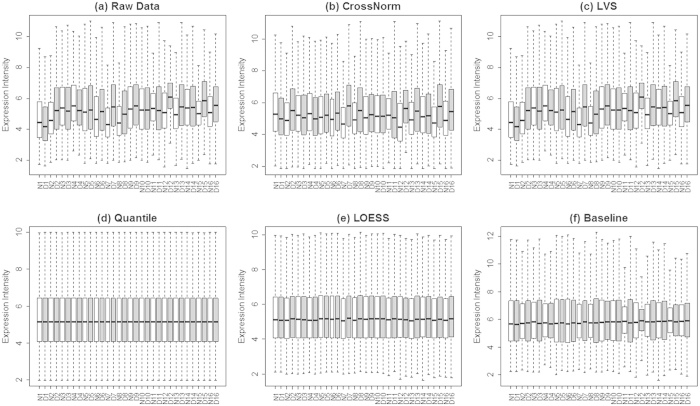
Boxplot of expression intensity of each sample for dataset Pancreatic32 before (**a**) and after (**b–f**) normalization. Samples in normal and disease group are represented by white and gray, respectively. Expression intensities were averaged over all samples of each group. The box stretches from the lower hinge (defined as the 25th percentile) to the upper hinge (the 75th percentile) and the median is shown as a line across the box.

To give a comprehensive assessment of the performance of CrossNorm, a total of four methods, including three conventional methods (Quantile, Baseline and LOESS) and the LVS relying less on the conventional assumptions, were employed for comparison. Firstly, we provided an overview of the global signal distribution between cancer and normal groups for the pair-matched cancer datasets normalized by each method. Figure 1a shows, in the pair-matched dataset Pancreatic32, genes of raw data have already been shown to be expressed much higher globally in cancer group than in the normal one^6^, but the clear change was removed by the three conventional normalization methods (Fig. 1d–f). The proportion of DEGs is expected to be low so that the per sample distributions of expression values are similar or even identical in these methods. For LVS, the trend was to some extent maintained, but it requires pre-selection of data driven features in an arbitrary proportion with the smallest array-to-array variation. When it comes to CrossNorm, however, it preserves the transcript changes between states while simultaneously processes all the arrays to remove system variation among them. Figure 1b shows that the overall expression increase from normal to cancer in the raw data can still be observed via data processed by CrossNorm. The same trend can also be detected using the other datasets listed in Table 1 (data not shown).

### Performance on simulated and spike-in dataset

**Figure 2:**
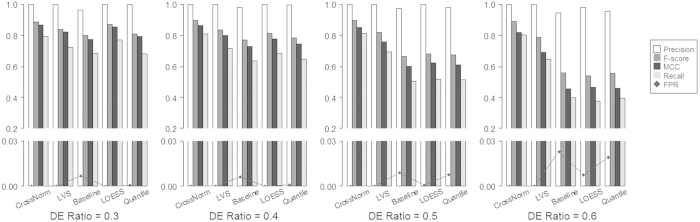
Impact of DEG ratio on the performance of CrossNorm, LVS, Baseline, LOESS and Quantile normalization on the simulated data. White, dark gray, black and light gray bars represent the measure of precision, F-score, MCC and Recall, respectively, while black diamond stands for FPR (False Positive Rate). The FPRs of the identified DEGs are consistently low when using CrossNorm and LVS across all DEG compositions, but it rises sharply with the increased DE ratio for the other three methods.

The performance of the CrossNorm method was firstly evaluated using two simulated datasets (ESCC34 and ESCC106) with specific proportions of up and down regulated Differentially Expressed Genes (DEGs, e.g., log_2_FC = ±0.8, ±1.0, and ±1.2) as mentioned in the Method section. The percentage of down-regulated DEGs was constant (10%) for all the compositions. For example, 20% are up-regulated while 10% are down-regulated, when the proportion of DEGs is predefined as 30%. The criteria of log_2_FC greater than 0.8 and P value of t-test less than 0.01 were used for detecting DEGs (Methods and datasets). Figure 2 shows the results of several measures for datasets with a series of DEG percentages from 20% to 50% as described in the section of Method and datasets. It is clear that CrossNorm consistently has the highest scores in all measures over all the scenarios for the simulated data ESCC34. Specifically, the precision is as high as or extremely close to 1.00 for all these methods when the Differential Expression (DE) ratio is 20% or 30%. For the conventional methods, however, the precisions drop obviously when the DE ratios are increased to 40% and 50%. For instance, the precisions are 0.9799, 0.9532 and 0.9454 when DE ratio is 50% for Baseline, LOESS and Quantile expression values, respectively. On the other hand, the recall and F-score of CrossNorm are around 0.8 and 0.9 while the measures are about 0.7 and 0.8 for the LVS normalization regardless of the feature compositions. For LOESS and Quantile, the measures of recalls and F-scores are less than CrossNorm but comparable to each other; all of them drop from around 0.65 and 0.8 to just 0.4 and 0.55 when the DE ratio is increased from 20% to 50%. Baseline is the most sensitive method to the DE ratios, all the measures of which decrease significantly with the increasing DE ratio. Furthermore, the False Positive Rates (FPRs) of the identified DEGs are consistently less than 0.0002 when using CrossNorm and LVS across all situations. This measure is much higher for the other three methods but still acceptable (less than 0.01) when DE ratio is less than 30%. However, the FPR rises sharply when the DE ratio is increased to 50%, which are 0.0077, 0.0193 and 0.0229 for Baseline, LOESS and Quantile, respectively. The same trend is also observed for the other simulated data ESCC106. To sum up, CrossNorm and LVS, both of which are not based on the strong assumptions, consistently return more reliable results while CrossNorm outperforms all the others for datasets with global and unbalanced biological variation.

**Figure 3:**
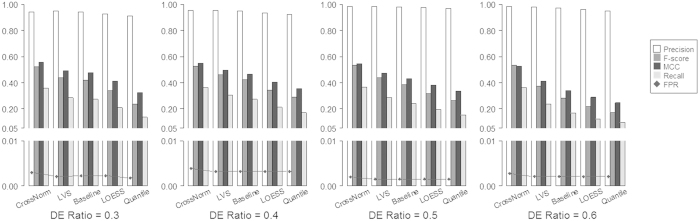
Impact of DEG ratio on the performance of CrossNorm, LVS, Baseline, LOESS and Quantile normalization on the spike-in data. White, dark gray, black and light gray bars represent the measure of precision, F-score, MCC and Recall, respectively, while black diamond stands for FPR (False Positive Rate). The FPRs are consistently less than 0.005 for all the methods regardless of the DEG compositions.

Spike-in data, which consist of probe sets with intensities for a gene spiked in at different known concentrations, are the perfect reference for evaluating the performance of normalization methods. The so-called Golden Spike-in data on Affymetrix DrosGenome1 platform^23^ was employed in this study, because it has imbalanced proportion of up-regulated genes. As demonstrated in Fig. 3, it is apparent that the performance of CrossNorm is better than the others. The measures of recall and F-score for CrossNorm are approximately 0.37 and 0.53 regardless of the DE ratios and are consistently higher than the other methods, which indicates that CrossNorm is not as sensitive as the conventional normalization methods to the DEG compositions. The precisions of CrossNorm also are higher than the three conventional methods across all the DE ratios, although they are a bit lower than that of LVS. For instance, the values are 0.9427 for CrossNorm while 0.9494 for LVS when the DE ratio is 0.2. On the other hand, the FPRs are consistently low for all the methods regardless of the DEG compositions, although it is higher for CrossNorm than that of the other methods. Overall, CrossNorm tends to detect more biological variations at the cost of having a slightly higher but acceptable FPRs.

### Performance on titration experiments

**Figure 4:**
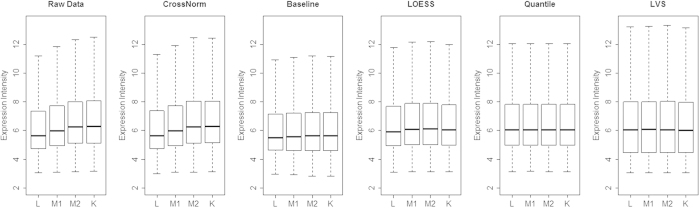
Expression value distributions for all probe sets averaged per mixture. L and K represent of lung and kidney while M1 and M2 stand for different mixtures of the two tissues, respectively.

The titration experiments were then employed to further evaluate the performance of CrossNorm. In contrast to spike-in studies where a set of transcripts are added at predetermined concentrations to some samples, titration series are not based on synthetic transcripts but they provide measurements from real-life biological samples that reflect the intricate characteristics of RNA samples. Although we do not know the authentic DEGs, the relationship between mRNA amounts throughout the titration series can be investigated and compared on measurements acquired from several normalization methods. As illustrated in Fig. 4, exploratory investigation of the raw data has revealed a distinct overall trend of expression intensities; the non-normalized expression intensities are broadly stronger in the kidney than in the liver samples. Arrays with a higher concentration of mRNA from the kidney samples are expected to produce higher expression values. The normalized expression values of the entire probe sets are not able to illustrate the overall expression increase from the liver to the kidney except the one normalized by CrossNorm. In other words, the overall increased trend in raw expressions can only be well detected by the CrossNorm method. Baseline and LOESS to some extent retain the trend but not as pronounced as CrossNorm. LVS and Quantile perform the worst for the mixture data and the expression distributions for both of them are more or less identical and hence no increase trend can be found.

**Figure 5:**
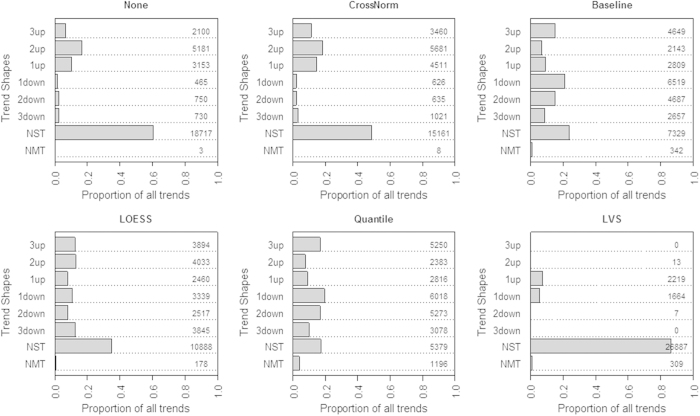
Shape analysis of the distribution of detected trends for the six normalization methods. NMT bar represents the percentage of non-monotonous trends and NST bar shows that of non-significant trends as described in the Method section. The other bars illustrate the percentages of genes showing one, two or three significant change(s) in up or down direction. The exact gene numbers are shown in the right hand side of the panels.

Figure 5 illustrates the observed proportions of significant trends. Data normalized by different methods shows distinct trend shapes of the category distribution (described in Methods and datasets). Klinglmueller, L. *et al.*^22^ found that data without any normalization shows approximately five times upward trends more than downward ones (bars marked up and down in Fig. 5) whereas these trends are more balanced for the Quantile and Baseline normalized data. Similar results were reported for the data normalized via the two methods as well as LOESS and LVS in this study. Surprisingly, CrossNorm normalized data illustrates approximately six times more significant upward than downward trends, which is highly consistent with the overall upward trend of expression values observed in Fig. 4. Furthermore, non-monotonous trend (NMT) is a clear indication of data artifacts, which is not expected to be detected in the titration series. However, we observed that all of the four other methods produce a huge number of NMTs, which are contrary to the experiment implications. Specifically, 342, 178, 1196 and 309 NMTs are observed in the data normalized by Baseline, LOESS, Quantile and LVS, respectively, whereas merely 3 and 8 NMTs are detected in the non-normalized and CrossNorm preprocessed data. Overall, CrossNorm performs quite comparable as the non-normalized data in artifact elimination and it can identify even more upward trends in the trend shape analysis.

### Effect on DEG identification and expression direction in cancer data

To further confirm the effectiveness of the CrossNorm method, we also investigated the reliability of solely identified DEGs for the cancer datasets with significant increases in the raw signal intensities in the cancer samples. As shown in Table 2, we compared the expression directions of the DEGs detected via CrossNorm and two other normalization methods, Quantile and LVS, for dataset ESCC106. Here, the expression direction of a gene represents the over-expression or down-expression of this gene in cancer samples compared with normal ones. The results illustrate that LVS and CrossNorm are more powerful than Quantile normalization in detecting DEGs. Specifically, 2889 and 2272 DEGs were detected when employing LVS and CrossNorm, respectively, but the number was decreased to 1843 when the dataset was processed by Quantile. When comparing Quantile and LVS, CrossNorm exclusively identified 693 and 811 genes as up-regulated DEGs, respectively. 5.63% of the 693 up-regulated DEGs that were solely selected by CrossNorm were listed as cancer genes in the Cancer Gene Census database. This was significantly higher than the corresponding proportion (3.74%) of background genes that are defined as all genes measured on the array (P = 0.007, hypergeometric test). In contrast, the proportion (3.03%) of down-regulated DEGs that were selected solely by Quantile was no higher than the background genes (P = 0.7762, hypergeometric test). Similarly, 5.67% of the 811 up-regulated DEGs that were exclusively identified using CrossNorm were in the cancer genes set, which was also significantly higher than the corresponding proportion of all background genes (P = 0.003, hypergeometric test). But for the down-regulated DEGs solely detected by Quantile, the proportion (2.32%) was much lower than the background genes. We can draw the same conclusion for dataset Pancreatic32 and Pancreatic78. Although no significance was identified for the other data ESCC34, the cancer gene ratio for the DEGs solely detected by CrossNorm (3.48%) is much higher than that of LVS (2.86%). These results indicate that the DEGs detected by CrossNorm for cancer datasets are more likely to be associated with cancer than using other methods.

Additionally, 78.79% (1790/2272) of the DEGs selected using CrossNorm were up-regulated, whereas the percentages for Quantile and LVS were only 59.52% (1097/1843) and 55.35% (1599/2889), respectively. Similar results can also be observed by using LOESS and Baseline. In the context of cancer cells, where genes tend to express higher, it is apparent that Quantile and LVS may more likely make an incorrect directional decision. This in turn indicates that CrossNorm is able to precisely identify DEGs and thereby provide more reliable interpretations for experiments.

### General CrossNorm performs comparably to Pairwise CrossNorm

For the Pairwise CrossNorm method, samples of the processed datasets should have pairwise relation between groups, and then it can normalize the data by assigning the pairwise samples as a new array. For non-pair datasets, on the other hand, the General CrossNorm method could also acquire a comparable result. In order to evaluate whether the performance of General CrossNorm is as powerful as Pairwise CrossNorm, we applied General CrossNorm and Pairwise CrossNorm on the simulated data ESCC34. As shown in Table 3, it is clear that the detected DEGs by the Pairwise and General CrossNorm are highly consistent. For instance, 2097 genes were selected with differential expression after the General CrossNorm normalization while 2074 out of them were also identified as DEGs via Pairwise CrossNorm for the data with 20% assigned DEGs. The results by the two methods were extremely consistent with the Overlapping Coefficient not less than 99% for all the scenarios. Hence, for expression datasets without the pairwise case-control relation, comparable results could also be produced by the General CrossNorm methods.

Moreover, General CrossNorm has acceptable computational efficiency. When we performed General CrossNorm on a reasonably sized experiment with 15,000 genes as well as 100 normal and 100 disease samples, which led to a 10,000 columns cross-matrix to normalize, it only costs 95 second on a 64-bit personal computer with Intel Core i5 3470 CPU @ 3.20GHz under the Windows operating system. When the sample size decreased to 50 vs 50 for normal and disease groups, which is a more normal case, the computation time dropped to approximately 30 second. Hence, CrossNorm in general is a fast, simple and efficient normalization method.

## Discussion

Theoretically, a global shift in gene expression occurs in cancers, as the alterations of many essential cellular functions collectively dictate malignant growth for practically all types of human cancers^24^,^25^. In practice, the amplification of gene expression levels during cancer development was also well documented via several works^6^,^8^,^10^. Hence blindly normalizing arrays to have similar distributions of probe intensities regardless of the sample condition may take a rather high risk of resulting in erroneous interpretations, although it is widely used in other studies^26^,^27^. Here we have proposed CrossNorm as a better alternative to existing normalization methods to process microarray experiments that contain global shifts over samples. The CrossNorm strategy has been demonstrated to have clear advantages by using simulated data, spike-in data, titration experiments and comprehensive real-life expression cancer datasets in comparison to three conventional global normalization methods and the LVS normalization that relies less on the assumption.

Our results have illustrated that conventional normalization methods tend to reverse the regulation direction of a large fraction of genes in cancer microarray data and the LVS algorithm might also over-normalize signals to a certain extent, while CrossNorm is able to take full advantage of the raw signal and more accurately estimate the regulation direction. As described in previous works and Jakob Love´n’s recent study^6^,^7^,^10^, many up-regulated DEGs associated with cancers were missed and more down-regulated ones were falsely produced when processed by global normalizations. The identification of the regulation direction of genes is also of vital importance for the subsequent biological analysis, as they play a critical role in studies like expression correlation of gene productions and regulation relations between transcript factor/miRNA and target mRNA, or merely the detection of the regulation direction of oncogene and tumor suppress genes^28^. All of these sorts of studies could be misled by inappropriate normalization methods. Besides, CrossNorm fully utilizes biological signals from the raw data rather than artificially presetting parameters with high impact specifically on the analysis, e.g., predefining a proportion of housekeeping genes in the LVS method. Also, the application of CrossNorm is very flexible. It is not restricted to cancer study, but also applicable to researches such as comparing tissues and developmental stages, as genes are expected to have high variation in both cases.

It is worth noticing that the expression values in samples in the treatment group may be shifted to some extend due to technical reasons. CrossNorm is on the basis that technical biases are independent of the treatment groups and it is not quite effective in eliminating such kind of artifact. In this paper, however, all the samples are required from the same experiment, so no or little batch effect exists between the compared sample groups. Also worth mentioning is that normalization methods are not very effective for the batch effect adjustment even for the Quantile, which forces all the arrays to have the same signal intensity distribution. However, it is still quite an issue being debated. Therefore we highly recommend the users to make sure that all the collected samples are from the same experiment batch before data normalization.

This consideration of gene’s behavior in biological conditions gives us a more comprehensive insight into interpreting the biological variance. In conclusion, CrossNorm is a robust and unbiased procedure that could help us better understand the expressional difference in a specific circumstance. An increasing number of genomic data detecting mRNA signal by different sorts of frameworks^29^,^30^ have been made available and opens new doors for investigation. To reduce the burden of data normalization, it is highly recommended to optimize experimental designs, stringently randomize potential experimental artifacts across biological groups and collect samples of sufficiently large sizes^6^. Also, it is worth noting that pairwise information between biological groups is of vital importance and more effort should be made for microarray preparation.

## Methods and Datasets

### Overview of the preprocessing procedure

Typically, the preprocessing procedure of microarray data consists of three steps: background correction, normalization and summarization. In the experiments, for uniformity, the raw data for each dataset were firstly processed using the RMA algorithm for background adjustment. Then, each probe-set ID was mapped to the official gene symbol. For a gene represented by multiple probe sets, we averaged its signal intensity in a sample for all the probe sets. Finally, the summarized data were processed separately via a series of methods for normalization, namely Quantile, Baseline (median scaling), LOESS, LVS and CrossNorm, respectively. Quantile, Baseline and LOESS normalization are well accepted methods and default values were applied for each of them. Quantile normalization is typically used by the Robust Multichip Average (RMA) while Baseline is a global normalization method that scales the expression values in each array with respect to a predefined baseline value. LVS defines a set of genes (t) with a low variation across all of the arrays and then uses a non-linear model to fit the genes from individual arrays to those from a reference array. It requires the pre-selection of a proportion of genes as a reference set. The suggested value of t is 60% or 40% by the authors. Here, we set t to 40%. Therefore, overall four categories of normalization method are utilized for comparison: rank-based model, baseline transformation, linear fitting model and data-driven housekeeping gene model. Quantile, Baseline, LOESS and LVS are the typical example for each normalization model, respectively.

### The procedure of Cross Normalization (CrossNorm)

**Figure 6:**
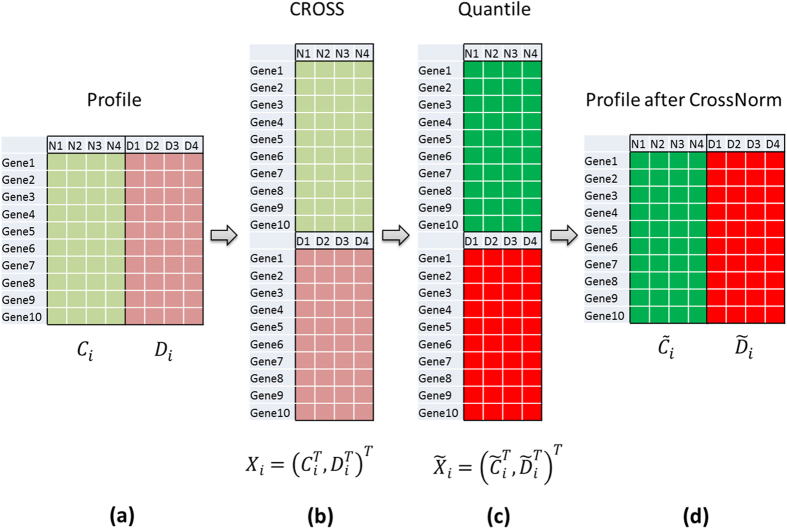
The flowchart for Pairwise CrossNorm normalization. (**a**) A profile to be normalized with pairwise normal (C_i_) and disease (D_i_) samples; (**b**) Cross Profile: reassemble the disease and normal profiles by column corresponding to their pairwise relation; (**c**) perform Quantile on the Cross Profile; (**d**) resume the positions of the disease and normal profiles.

CrossNorm consists of two versions, Pairwise CrossNorm and General CrossNorm, depending on whether the datasets have matching pairwise relation between conditions or not, as it is quite restrictive to require all the expression experiments to have pairwise case-control tissue samples. It is very flexible and easily generalizable to all the prevalent normalization procedures. For brevity, CrossNorm represents Pairwise CrossNorm and is a modification of Quantile in the present paper unless explicitly stated. The flowchart shown in Fig. 6 illustrates the Pairwise CrossNorm procedure. It is advised to process the raw data using a particular background correction method, execute the probe set summarization, and then perform CrossNorm.

Let 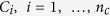 be the expression profiles of the 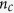 control arrays, and let 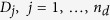 be the expression profiles of the 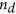 disease arrays. The 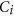 and 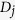 expression profiles have the same length (the number of genes) *m*. We assume that the 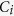 and the 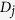 expression profiles are independent identically distributed (i.i.d.) for both paired and unpaired datasets.

In a paired dataset, where 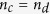, Pairwise CrossNorm is performed as follows:

- Form a matrix of 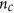 columns 
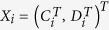
 (i.e. concatenate the two column vectors), for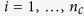.
- Normalize the columns 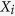 using an appropriate approach, such as Quantile, to obtain a matrix with columns
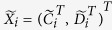
; obtain the final normalized control cases as 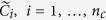, and the normalized disease cases as 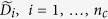.

Furthermore, a General CrossNorm is defined for the unpaired datasets. Its workflow is as follows:

- Form a large matrix with 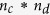 columns 
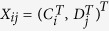
, for 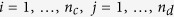; normalize the columns 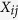 using an appropriate approach (Quantile in this study) to obtain a matrix with columns 
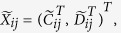
where both 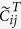 and 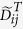 are of length *m*.
- Obtain the final normalized control cases as 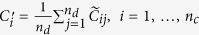, and the normalized disease cases as 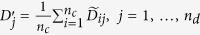. 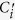 is the average of the elements of the normalized columns originally formed from 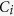, and similarly for 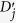.

The Quantile normalization requires all arrays in the same distribution; therefore, we argue that each column of the column-binding matrix retains the same distribution. When the data are paired, 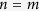, 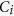 and 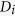 originate from the same individual, corresponding to the expressions of the normal and disease cells, respectively. 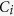 and 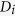 may be dependent, but since the data are for the same disease, we may assume that the dependence is similar in different patients. Therefore we may assume that 
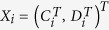
, 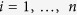 are i.i.d. For an unpaired dataset, we merge the control and disease arrays by forming a large matrix where the columns are 
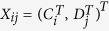
. Because the 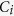 expression profiles are i.i.d. and are independent of the 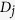 expression profiles, 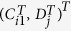 and 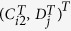 have the same distribution and are dependent. Similarly, 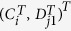 and 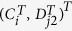 are dependent and have the same distribution. Therefore, 
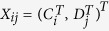
 for 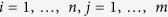 all have the same distribution, though some columns may be dependent.

In either case, we may assume that the columns of the merged expression matrix have the same distribution. Therefore, it is reasonable to perform the Quantile normalization on the cross-matrix to obtain a comparable expression profile. In addition, the current implementation of CrossNorm can normalize the expression data at either the probe or the probeset level.

### Microarray gene expression datasets

From the NCBI GEO database^13^, we collected the Affymetrix^23^ datasets with pair-matched cancer and normal samples according to the following criteria: 1) each dataset must consist of at least 20 arrays (10 for each condition) and all of which are from the same platform, and 2) the expression level increases (marginally) significantly in cancer state. Ultimately we collected a total of 10 datasets across 8 cancer types with all samples being pair-matched, all of which are listed in Table 1^6^,^7^. For datasets with pair-matched cancer and control samples, the effects of certain complex factors, such as familial, individual and environmental differences can be avoided and hence more reliable signals can be produced^6^.

### Simulation, spike-in and titration series data

Three types of data, simulation, spike-in and titration experiment, were used to evaluate and compare the performance of CrossNorm with those of other normalization algorithms in this study.

For the simulated data, in order to retain the intrinsic structure of the data, data were simulated for 34 disease samples and 106 disease samples based on the expression profiles of 12,752 genes for 34 and 106 normal esophagus tissue samples extracted from the GSE20347 and GSE23400 datasets, respectively^31^. For normal samples, a proportion of DEGs were produced and used to produce a disease sample by setting these genes with different magnitudes of differential expression (e.g., log_2_FC = 0.8, 1 and 1.2). The mean vector for each gene in the disease sample group was determined by sampling from a Gaussian distribution whose mean was equal to the corresponding normal group mean and variance was the same as original disease group. Random noise sampled from a chi-squared distribution was added to each means. Eventually, this dataset was simulated to have two groups with the same sample size.

Spike-in dataset is produced by controlled experiments with known RNA concentrations and assigned Fold Change (FC) before detection. The spike-in DrosGenome1 dataset^23^ designed for group comparison provides a dataset of 14,010 probe sets, 3,866 of which are assigned concentration folds. Specifically, 2,535 of them had been assigned unchanged concentrations, namely FC equal to 1, while 1,331 with FC greater than 1. The other empty probe sets were not spiked any concentration. To produce an expression profile with a specific percentage of differentially expressed genes (DEGs), gene products with priori concentrations fold greater than a given threshold (actual DEGs) are involved while the non-DEGs are selected from both the unchanged and empty probe set pool. For example, the produced profile has 4,437 genes when the assigned DE ratio is 0.3, consisting of all the 1,331 probe sets with assigned higher FC and the 3,106 others with unchanged or unknown concentration fold. Each group consists of 3 replicate arrays for both spike-in datasets and eventually profiles with 6 arrays were laid out.

For the titration series data, the EMERALD experiment was used, in which the total RNA was extracted from liver and kidney tissues of six rats^22^. The resulting sample material was then composed in four mixtures: (L) pure liver material; (M1) 75% liver and 25% kidney material; (M2) 25% liver and 75% kidney; and (K) pure kidney material. Since equal mRNA amounts were used for each array, the produced signal intensities merely reflect the fraction constituted by mRNA. Titration series provide measurements from real-life biological samples that reflect the intricate characteristics of RNA samples, but no ground truth is provided. Namely, we do not know the exact FC for each probe and therefore which genes are authentically differentially expressed. The only prior knowledge available in titration series experiments is the mixture proportions and the relationship between mRNA amounts over the titration series. The Affymetrix platforms used in this study for the EMERALD project is Rat Genome 230 2.0. Each mixture group consists of 24 arrays.

### DEGs identification and consistency statistic

One of the main aims of normalization is differential analysis across samples. Results from the MAQC project^32^ indicate that a straightforward approach of FC ranking plus a non-stringent P value threshold can be successful in identifying concordant gene lists, whereas merely selecting DEGs via the t-test statistic predestine a poor reproducibility in results, because of the relatively unstable nature of the variance (noise) estimate in the t-statistic measure. The same as the MAQC project, both expression FC with a threshold and t-test with a P value were applied for detecting DEGs. The criteria were Fold Change (log_2_FC) greater than or equal to 0.8 and P value less than 0.01. All the calculations are on log2 scale.

We calculated both the Overlap Coefficient (OC) and Direction Overlap Coefficient (DOC) ratio to assess the consistency of two given gene sets. OC is defined as


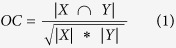


where X and Y represent the two detecting gene sets, respectively. Similarly, DOC is the ratio of the genes that have the same regulation direction in both gene sets. DOC1 (or DOC2) is the percentage of the DEGs in set 1 (or in set 2) regulating in the same directions in another dataset from which the DEGs in set 2 (or in set 1) were extracted.

### The Evaluation of differential analysis

Precision, Recall, False Positive Rate (FPR), F1-score and Matthews Correlation Coefficient (MSS) are employed in this study to measure the performance of each normalization method. The spike-in experiment enables us to know the true DEGs a priori, which facilitates us to compute all of these measures. Here, the precision is defined as the ratio of correctly identified DEGs to all detected DEGs and the recall is defined as the ratio of correctly identified DEGs to all true DEGs. The F1-score is a harmonic mean of precision and recall and its formula is:


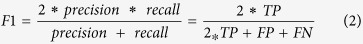


As recommended by MAQC II^33^, we also report performance based on Matthews Correlation Coefficient (MCC) because it is informative when the distribution of the two classes in a dataset is highly skewed and it is simple to calculate and available for all models. The MCC value is more useful than other measurements, such as ROC curve, since by definition a ROC curve is constructing the performance over all possible cutoffs. But in the case of differential analysis, only one or a few reasonable cutoffs are provided for identifying DEGs. MCC values range from –1 to 1 with 0 indicating random prediction. −1 and 1 indicate total inverse prediction and perfect prediction, respectively. MCC can be calculated directly as follows:


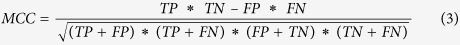


TP, TN, FP, and FN represents true positive, true negative, false positive and false negative, respectively.

### Measures for the titration experiment

In the titration experiment, the difference between adjacent mixtures (L−M1, M2−M1 and K−M2) can be positive, negative or no difference. So totally 27 changes could be detected for each feature and these changes were ultimately categorized into eight types of trends, significant non-monotonous trend (NMT), non-significant trend (NST) and monotonous trend characterized by the number of significant changes, 1up, 2up and 3up for the upward trend and 1down, 2down and 3down for the downward trend, respectively. Significant non-monotonous trend is defined as at least one significant increase together with at least one significant decrease while non-significant trend indicates no significant expression changes. The detailed information of the method for trend test is described in *Klinglmueller, F. et al.*^22^. The measures can be calculated using R package orQA, which is available in CRAN http://cran.r-project.org.

## Additional Information

**How to cite this article**: Cheng, L. *et al.* CrossNorm: a novel normalization strategy for microarray data in cancers. *Sci. Rep.* **6**, 18898; doi: 10.1038/srep18898 (2016).

## Supplementary Material

Supplementary Information

## Figures and Tables

**Table 1:** Microarray gene expression datasets with paired samples.

Dataset	Accession Number	Platform	Disease Name
Breast26	GSE10780	HG-U133_Plus_2	Breast cancer, Invasive Ductal Carcinoma (IDC)
Colon34	GSE18105	HG-U133_Plus_2	Colorectal Cancer (CRC)
ESCC34	GSE20347	HG-U133A_2	Esophageal Squamous Cell Carcinoma (ESCC)
ESCC106	GSE23400	HG-U133A	Esophageal Squamous Cell Carcinoma (ESCC)
Gastric62	GSE13911	HG-U133_Plus_2	Primary Gastric Tumors
HCC20	GSE29721	HG-U133_Plus_2	Hepatic Cellular Carcinoma (HCC)
HNSCC44	GSE6631	HG_U95Av2	Head and Neck Squamous Cell Carcinoma (HNSCC)
OTSCC40	GSE13601	HG_U95Av2	Oral Tongue Squamous Cell Carcinoma (OTSCC)
Pancreatic32	GSE16515	HG-U133_Plus_2	Pancreatic Tumor
Pancreatic78	GSE15471	HG-U133_Plus_2	Pancreatic Ductal Adenocarcinoma

The name of each dataset follows the simple naming pattern: cancer type followed by sample size. In total 12 datasets were collected.

**Table 2:** The impact of normalization on the regulation directions of DEGs in the ESCC106 dataset.

DEGs	Quantile	LVS	CrossNorm	Quantile vs. CrossNorm			LVS vs. CrossNorm		
				Quantile exclusive	CrossNorm exclusive	Common genes	LVS exclusive	CrossNorm exclusive	Common genes
Up-regulation	1097	1599	1790	0	693	1097	620	811	979
Down-regulation	746	1290	482	264	0	482	818	10	472
Total	1843	2889	2272	—	—	1579	—	—	1451

**Table 3:** Statistic of DEGs identified after Pairwise and General CrossNorm for simulated data ESCC34.

Assigned DE ratio	No. of detected DEG Pairwise CrossNorm	No. of detected DEG General CrossNorm	Overlap genes	Overlapping Coefficient
0.2	2087	2097	2074	99.14%
0.3	3120	3145	3111	99.31%
0.4	4086	4115	4075	99.38%
0.5	5191	5222	5175	99.41%

DEG: Differentially Expressed Gene.
