# Supplementary material for: CrossNorm: a novel normalization strategy for microarray data in cancers
Source: Sci Rep. 2016 Jan 6;6:18898. doi: 10.1038/srep18898 (PMC4702063; doi:10.1038/srep18898)
Supplement: Supplementary Information [file srep18898-s1.pdf]

CrossNorm: a novel normalization strategy for microarray data in cancers

Lixin Cheng[1], Leung-Yau Lo[1], Nelson LS Tang[2,] *, Dong Wang[3,]*, Kwong-Sak Leung[1,]*

[1]Department of Computer Science and Engineering, The Chinese University of Hong Kong, Shatin, New Territories, Hong Kong, [2]Department of Chemical Pathology, The Chinese University of Hong Kong, Shatin, New Territories, Hong Kong, [3]College of Bioinformatics Science and Technology, Harbin Medical University, Harbin, China.

* Correspondence and requests for materials should be addressed to

K.L. (ksleung@cse.cuhk.edu.hk) or D.W. (wangdong@ems.hrbmu.edu.cn) or N.T. (nelsontang@cuhk.edu.hk)

```
#===================================================================================
# Description:
#      Cross Normalization (CrossNorm) for gene expression data.
#
# Arguments:
#      exp  -  a (non-empty) numeric matrix of data values. Row represents gene while
#               colum represents sample.
#      label - a (non-empty) binary vector of data values in which ’0’ represents
#               control sample and ’1’ reptesents disease sample. The length of label
#               should be equal to the column number of exp.
# Value:
#      exp.crossnorm - A normalized numeric matrix. Row represents gene while column
#                      represents sample. The gene order is the same as exp.
#
# Reference:
#      CrossNorm: a novel normalization strategy for microarray data in cancers
#      Lixin Cheng, Leung-Yau Lo, Kwong-Sak Leung, Nelson LS Tang and Dong Wang
#
# Example:
#      source("CrossNorm.R")
#      exp.pcn = PairedCrossNorm(exp, label)
#      exp.gcn = GeneralCrossNorm(exp, label)
#===================================================================================

library(affy)
library(preprocessCore)

# -------------------Paired CrossNorm --------------------

PairedCrossNorm <- function(exp, label){
       exp = as.matrix(exp);
       geneLen = dim(exp)[1];
       exp.normal = exp[,label==0];
```

```
        exp.disease = exp[,label==1];
        exp.cross = rbind(exp.normal,exp.disease);
        exp.quantile.cross = normalize.quantiles(exp.cross);
        exp.crossnorm.normal = exp.quantile.cross[1:geneLen,];
        exp.crossnorm.disease = exp.quantile.cross[(geneLen+1):(2*geneLen),];
        exp.crossnorm= cbind(exp.crossnorm.normal,exp.crossnorm.disease);
        return(exp.crossnorm)
}

# ---------------- General CrossNorm --------------------

GeneralCrossNorm <- function(exp,label){
        exp = as.matrix(exp);
        exp.cross = Matrix2CrossMatrix(exp,label)
        exp.quantile.cross = normalize.quantiles(exp.cross)
        exp.crossnorm = CrossMatrix2Matrix(exp.quantile.cross,label)
        return(exp.crossnorm)
}

# CrossMatrix
Matrix2CrossMatrix <- function(M, label){
 M = as.matrix(M)
 rowLen = dim(M)[1]
 sampleSize1 = sum(label==1) # disease sample size
 sampleSize0 = sum(label==0) # normal sample size

 indexMatrix = matrix(1:(sampleSize1*sampleSize0),,sampleSize0)
 M1 = M[,label==1]
 M0 = M[,label==0]
 M3 = matrix(0,rowLen*2,sampleSize1*sampleSize0)
 for (t in 1:sampleSize1){
        M3[,indexMatrix[t,]] = rbind(matrix(rep(M1[,t],sampleSize0),,sampleSize0),M0)
 }
 return(M3)
}

CrossMatrix2Matrix <- function(CrossM,label){
 rowLen = dim(CrossM)[1]/2
 sampleSize1 = sum(label==1) # disease sample size
 sampleSize0 = sum(label==0) # normal sample size

 indexMatrix = matrix(1:(sampleSize1*sampleSize0),,sampleSize0)
 M1 = matrix(0,rowLen,sampleSize1)
 M0 = matrix(0,rowLen,sampleSize0)
 for(t in 1:sampleSize1){
  M1[,t] = apply(CrossM[1:rowLen,indexMatrix[t,]],1,mean)
 }
 for(t in 1:sampleSize0){
  M0[,t] = apply(CrossM[(rowLen+1):(rowLen*2),indexMatrix[,t]],1,mean)
 }
 M = cbind(M0,M1)
 return(M)
}
```
